# Reduced cardiac ^123^I-MIBG uptake is a robust biomarker of Lewy body disease in isolated rapid eye movement sleep behaviour disorder

**DOI:** 10.1093/braincomms/fcae148

**Published:** 2024-04-26

**Authors:** Tomoyuki Miyamoto, Masayuki Miyamoto

**Affiliations:** Department of Neurology, Dokkyo Medical University Saitama Medical Center, Saitama, 343-8555, Japan; Center of Sleep Medicine, Dokkyo Medical University Hospital, Tochigi, 321-0293, Japan; Graduate School of Nursing, Dokkyo Medical University, Tochigi, 321-0293, Japan

**Keywords:** dementia with Lewy bodies, Lewy body disease, Parkinson’s disease, REM sleep behaviour disorder, ^123^I-MIBG

## Abstract

Cardiac ^123^I-MIBG scintigraphy is used to assess the function of postganglionic presynaptic cardiac sympathetic nerve endings. ^123^I-MIBG cardiac uptake is markedly reduced in patients with isolated rapid eye movement sleep behaviour disorder, similar to Parkinson’s disease and dementia with Lewy bodies. As a result, it can be used as an early biomarker of isolated rapid eye movement sleep behaviour disorder. Most patients with isolated rapid eye movement sleep behaviour disorder develop synucleinopathies: Parkinson’s disease, dementia with Lewy bodies or multiple system atrophy. We aimed to investigate whether cardiac postganglionic denervation is present in patients with isolated rapid eye movement sleep behaviour disorder, as well as its possible usefulness as a marker for Lewy body disease status. This retrospective cohort study examined 306 patients (236 men and 70 women; mean age: 68.2 years; age range: 43–87 years) with polysomnography-confirmed isolated rapid eye movement sleep behaviour disorder who were followed for 1–3 months and underwent ^123^I-MIBG scintigraphy. We retrospectively analysed data from 306 patients with polysomnography-confirmed isolated rapid eye movement sleep behaviour disorder, and their longitudinal outcomes were documented at two centres. Among isolated rapid eye movement sleep behaviour disorder patients, reduced ^123^I-MIBG uptake was observed in the early and delayed images in 84.4 and 93.4% of patients, respectively, whereas 88.6% of the patients had a high washout rate. This large Japanese two-cohort study (*n* = 306) found that 91 patients (29.7%) developed an overt synucleinopathy (51 Parkinson’s disease, 35 dementia with Lewy bodies, 4 multiple system atrophy, and 1 cerebellar ataxia) during a mean follow-up duration of 4.72 ± 3.94 years, with a conversion risk of 14.5% at 3 years, 25.4% at 5 years, 41.4% at 8 years and 52.5% at 10 years. On the other hand, among patients with heart-to-mediastinum ratio < 2.2 in the delayed images (*n* = 286), 85 (29.7%) developed Parkinson’s disease or dementia with Lewy bodies during a mean follow-up duration of 4.71 ± 3.94 years, with a conversion risk of 14.5% at 3 years, 25.6% at 5 years, 42.0% at 8 years and 51.0% at 10 years. Among the 33 patients who underwent repeat ^123^I-MIBG scintigraphy, there was a progressive decline in uptake over the next 4.2 years, with patients exhibiting reduced uptake progressing to Parkinson’s disease or dementia with Lewy bodies. In contrast, patients without decreased ^123^I-MIBG uptake progressed to multiple system atrophy. Reduced cardiac ^123^I-MIBG uptake was detected in over 90% of isolated rapid eye movement sleep behaviour disorder patients, with progression to Parkinson’s disease or dementia with Lewy bodies, rather than multiple system atrophy, over time. Reduced ^123^I-MIBG uptake is a robust maker for Lewy body disease among isolated rapid eye movement sleep behaviour disorder patients.

## Teaser

Miyamoto and Miyamoto demonstrated reduced cardiac ^123^I-MIBG uptake as a robust marker of isolated rapid eye movement sleep behaviour disorder in >90% of cases. These patients are high risk for Lewy body disease. Reduced and normal uptakes were associated with progression to Lewy body disease and multiple system atrophy, respectively.

## Graphical Abstract

**Graphical Abstract:**
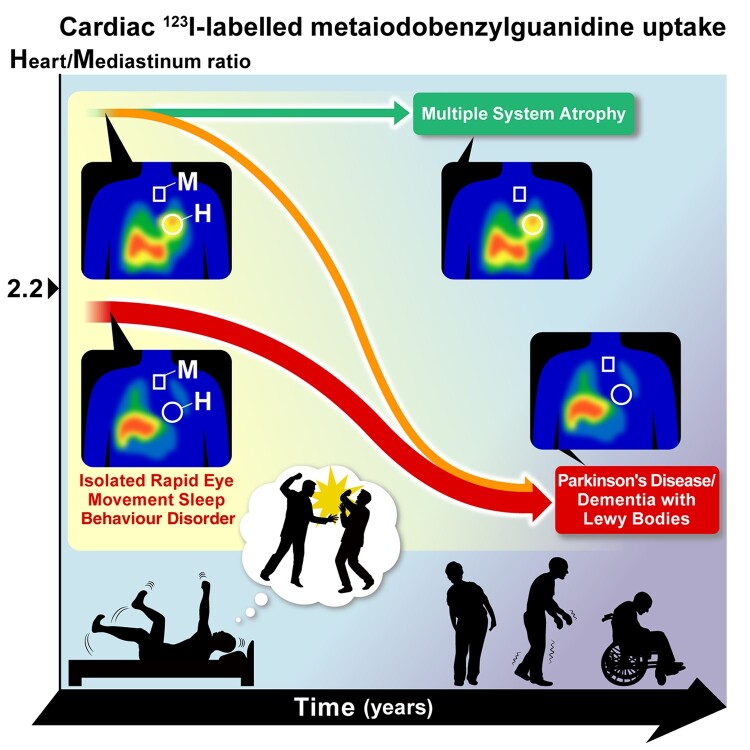

## Introduction

Isolated rapid eye movement sleep behaviour disorder (IRBD) is a sleep-associated disorder characterized by abnormal behaviour along with dreams during rapid eye movement (REM) sleep. It was first reported by Schenck *et al*.^1^ in 1986. In previous studies, 81–90% of IRBD patients developed a neurodegenerative disorder.^2^ Recent studies have suggested that IRBD is a precursor condition to distinct neurodegenerative changes. Longitudinal reports suggest that the rate of phenoconversion from IRBD to Parkinsonism or dementia increases over time.^3^

^123^I-Metaiodobenzylguanidine (^123^I-MIBG), a physiologic analogue of noradrenaline, is used to determine the location, integrity and function of postganglionic noradrenergic neurons. Cardiac ^123^I-MIBG scintigraphy can assess postganglionic cardiac autonomic denervation and has become widely used in Japan since its introduction to clinical cardiology and neurology practice in the 1990s.^4^ Neurological studies have found reduced cardiac ^123^I-MIBG uptake in Parkinson’s disease, dementia with Lewy bodies (DLB), pure autonomic failure, and Parkinson’s disease-related movement disorders.^5^ A multicentre study of the diagnostic performance of ^123^I-MIBG for differentiation between Alzheimer’s disease and DLB found a high correlation between abnormal cardiac sympathetic activity on ^123^I-MIBG cardiac scintigraphy and a clinical diagnosis of probable DLB. The diagnostic accuracy is sufficiently high for this technique to be clinically useful for distinguishing between DLB and Alzheimer’s disease, particularly in patients with mild dementia.^6^ A follow-up study revealed a higher correlation between abnormal cardiac sympathetic nerve activity assessed by cardiac ^123^I-MIBG scintigraphy and a clinical diagnosis of probable DLB compared to earlier cross-sectional studies. The results also suggested the usefulness of cardiac ^123^I-MIBG scintigraphy in the early clinical stages of DLB.^7^ On the other hand, severely reduced cardiac ^123^I-MIBG uptake in Parkinson’s disease patients is associated with a risk of developing subsequent dementia, suggesting a link between cardiac noradrenergic denervation and cognitive function, similar to DLB.^8^

In an autopsy-based case report of a patient with IRBD for 20 years, confirmed by polysomnography (PSG), incidental Lewy body disease was diagnosed, with Lewy bodies found in the locus coeruleus and substantia nigra.^9^ Furthermore, Boeve *et al*.^10^ investigated the neuropathological diagnosis of 15 patients with a history of IRBD (10 PSG confirmed) and found that 12 had Lewy body disease (11 neocortical and 1 peripheral) and 3 had multiple system atrophy (MSA). In 10 of 15 (66.7%) patients with IRBD, dementia or Parkinson’s disease manifested after a median of 10 (range 2–29) years. These findings suggest that the underlying cause of IRBD is abnormal α-synuclein. In IRBD, the affected areas include not only the brainstem regions regulating REM sleep but also regions such as the olfactory system, nigrostriatal system and autonomic system. Cardiac ^123^I-MIBG scintigraphy studies in IRBD patients with prodromal Parkinson’s disease or DLB status have shown significantly reduced uptake in IRBD patients compared to controls, suggesting post-synucleinopathy, but this finding is more common in Parkinson’s disease patients than in MSA patients^11^ and is considered a prodromal phenotype.^12^ In a meta-analysis of cross-sectional studies of IRBD, cardiac ^123^I-MIBG uptake was reduced in Parkinson’s disease and DLB to the same extent as in Lewy body disease.^13^ Although these biomarkers are diagnostic rather than prognostic, there are no reports of large-scale longitudinal data showing an association with phenotypic conversion rates.^14^ In this study, we retrospectively examined the cardiac ^123^I-MIBG uptake and outcomes of consecutive IRBD patients from two centres, as well as the changes in cardiac ^123^I-MIBG uptake over time in patients who underwent two or more cardiac ^123^I-MIBG tests.

## Materials and methods

**Figure 1:**
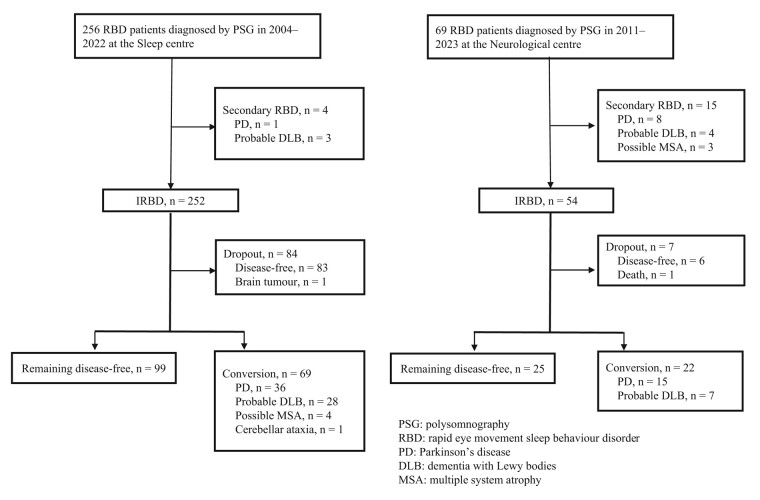
**Study flowchart for the follow-up of patients with IRBD.** IRBD, isolated rapid eye movement sleep behaviour disorder; PSG, polysomnography; PD, Parkinson’s disease; DLB, dementia with Lewy bodies; MSA, multiple system atrophy.

We retrospectively reviewed our database for consecutive patients with IRBD. This study included a total of 306 patients who were diagnosed with IRBD by PSG and underwent cardiac ^123^I-MIBG scintigraphy between August 2004 and July 2022 at the Sleep Medicine Centre of Dokkyo Medical University Hospital (sleep centre) and between August 2011 and July 2023 at the Saitama Medical Centre of Dokkyo Medical University (neurological centre; Fig. 1). IRBD was diagnosed on the basis of a history of dream-enacting behaviours and video PSG demonstration of increased electro-myographic activity during REM sleep that was associated with abnormal behaviours. These criteria are based on the second edition of the International Classification of Sleep Disorders. In the sleep centre, ^123^I-MIBG testing was performed on consecutive IRBD cases, whereas in the neurological centre, ^123^I-MIBG testing was performed on randomly selected IRBD patients. The cut-off value for early or delayed reduction in cardiac ^123^I-MIBG uptake was <2.2,^15^ and the degree of ^123^I-MIBG uptake and longitudinal prognostic outcomes were retrospectively analysed for the 306 patients. Repeated ^123^I-MIBG testing (*n* = 33) was performed only at the sleep centre, demonstrating normal uptake (≥2.2) on both early and delayed images or only on early images in 19 (57.6%) patients and reduced uptake (<2.2) on both early and delayed images at baseline in 14 (42.4%) patients. The association between changes in ^123^I-MIBG uptake and prognosis was also analysed in 33 IRBD patients.

After the scans were performed, all IRBD patients were systematically followed with neurological examinations and detailed clinical histories, including for cognitive and motor problems, every 1–3 months by a neurologist (T.M. and M.M.) with expertise in both sleep disorders and neurodegenerative diseases, applying diagnostic criteria for Parkinson’s disease,^16^ DLB^17^ and MSA.^15,18^ The findings were entered into the medical records of patients. The medical records were reviewed retrospectively between March and July 2023 (end of the current study) to assess the presence and nature of neurodegenerative diseases, such as Parkinsonism or dementia, identified during follow-up with the same neurologist (T.M. and M.M.). The motor component of the Unified Parkinson’s Disease Rating Scale-III was also administered, and the Folstein Mini-Mental State Examination was used to evaluate cognitive function, similar to previous studies.^19^

### ^123^I-MIBG cardiac scintigraphy

Early (15–30 min) and delayed (3–4 h) images were obtained after injection of 111 MBq ^123^I-MIBG (Daiichi Radioisotope Laboratories Co.; Fujifilm Toyama Chemical Industries; PDRadiopharma Inc., Tokyo, Japan). The scintigraphy findings were immediately interpreted and recorded. Photopeak energy was centred at 159 keV (^123^I-MIBG) with a window of 10%. The relative organ uptake of ^123^I-MIBG was determined by setting the region of interest on the anterior view (Supplementary Fig. 1). The heart-to-mediastinum (H/M) ratio was calculated using the standard method by dividing the mean number of counts per pixel in the circular region of interest of the heart by the mean number of counts per pixel in the rectangular region of interest of the superior mediastinum. Because of the differences in test timings and facilities where ^123^I-MIBG cardiac scintigraphy was performed, differences in collimator (GCA-7200, Toshiba; GCA-9300A-HG, Toshiba; SYMBIA E, Siemens Healthcare; E-CAM, Siemens Healthcare) were used for calibration phantoms or standardized using conversion factors established in previous studies.^20^ The H/M ratios were converted to values equivalent to those of medium energy collimators. The washout rate was calculated from early and delayed heart counts (*H*_early_ and *H*_delayed_, respectively) and mediastinal counts (*M*_early_ and *M*_delayed_, respectively), using the following formula for background (mediastinal counts) and decay corrections: ([{*H*_early_ − *M*_early_} − {*H*_delayed_ − *M*_delayed_} × *k*]/[*H*_early_ − *M*_early_] ×100) (%). The coefficient *k* is a time decay correction factor of 1/0.5*^t^*^/13^ for time *t* (h). If the interval between the scans is 3 h, *k* is 1.17. The washout rates were not standardized. The standard cut-off value was used for the washout rate (34%). None of our patients had taken drugs known to affect the ^123^I-MIBG uptake (e.g. tricyclic antidepressants, selective serotonin reuptake inhibitor and selegiline), and none had a history of cardiac disease or untreated diabetes mellitus. The normal H/M ratio for myocardial ^123^I-MIBG scintigraphy is ≥ 2.2 for the early and delayed images, whereas the normal washout rate is ≤34%.^20^

The study protocol was approved by the Ethics Committee of Dokkyo Medical University (approval number R-2-22) and the Ethics Committee of Saitama Medical Centre of Dokkyo Medical University (approval number 1821). The study was conducted in accordance with the ethical standards set out in the 1964 Declaration of Helsinki and subsequent amendments. Written consent was obtained from the participants, but because of the retrospective design, some patients were unable to give written consent and had the opportunity to opt out of the study at any time and seek further explanation.

### Statistical analysis

Descriptive demographic, clinical and neuroimaging data are presented as means with standard deviations (SDs) or numbers with percentages. Comparisons between groups were performed using the Kruskal–Wallis test. Neurological disease-free survival rates were estimated using the Kaplan–Meier analysis. Disease-free survival rates were assessed from the date of ^123^I-MIBG scintigraphy to the date of Parkinson’s disease, DLB or MSA diagnosis, or to the last follow-up for censored observations. Repeated measures paired *t*-test was used to evaluate the differences in ^123^I-MIBG uptake between baseline and repeat evaluations at 4.2 ± 2.8 years. A *P* < 0.05 was considered statistically significant.

All statistical analyses were performed using Prism (version 9 for Mac OS X; GraphPad Software, Inc., San Diego, CA, USA), SPSS (version 28.0; IBM Corp., Armonk, NY, USA) and R (version 4.0.1; freely available at https://www.R-project.org) software.

## Results

Overall, 306 IRBD patients, with a ^123^I-MIBG H/M cut-off of 2.2, showed reduced ^123^I-MIBG uptake in 260 (85.0%) early and 286 (93.5%) delayed images at baseline (Table 1).

**Table 1:** Demographic data of cardiac ^123^I-MIBG scintigraphy in patients with IRBD at baseline

	Sleep centre cohort between 2004 and 2022 (*n* = 252)	Neurological centre cohort between 2011 and 2013 (*n* = 54)	Combined sleep centre cohort and neurological centre cohort (*n* = 306)
Age at PSG, years	67.8 (7.3)	68.4 (8.1)	67.9 (7.4)
Sex			
Women	56 (22.2%)	14 (25.9%)	70 (22.9%)
Men	196 (77.8%)	40 (74.1%)	236 (77.1%)
RBD onset age (subjective), years	60.7 (9.6)	63.6 (9.3)	61.2 (9.6)
RBD duration (subjective estimated), years	7.1 (7.1)	6.6 (5.1)	7.0 (6.8)
RBD duration (diagnosis), years	1.0 (0.76)	1.4 (2.3)	0.3 (1.3)
MMSE total score	28.0 ± 2.5*	27.5 ± 2.7**	27.5 ± 3.1***
1987 UPDRS-III total score	2.0 ± 2.7****	3.4 ± 2.7*****	2.5 ± 2.7******
^123^I-MIBG scintigraphy			
Age, years	67.8 (7.3)	70.2 (7.9)	68.2 (7.4)
Interval between ^123^I-MIBG and outcome, years	4.3 (4.0)	3.1 (2.8)	4.7 (3.9)
Early H/M	1.86 (0.40)	1.66 (0.37)	1.82 (0.40)
Early H/M, <2.2	211/252 (83.7%)	49/54 (90.7%)	260/306 (85.0%)
Delayed H/M	1.52 (0.46)	1.49 (0.40)	1.52 (0.45)
Delayed H/M, <2.2	234/252 (92.9%)	52/54 (96.3%)	286/306 (93.5%)
Washout rate, %	50.6 (13.4)	22.2 (5.1)	45.6 (16.5)
Washout rate, ≥23%	241/252 (95.6%)	30/54 (55.6%)	271/306 (88.6%)

Data are mean (SD). H/M, heart-to-mediastinum; ^123^I-MIBG, ^123^I-metaiodobenzylguanidine; IRBD, isolated REM sleep behaviour disorder; MMSE, Mini-Mental State Examination; PSG, polysomnography; UPDRS, Unified Parkinson’s Disease Rating Scale. *n = 248; **n = 49; ***n = 297; ****n = 242; *****n = 54; ******n = 296.

**Figure 2:**
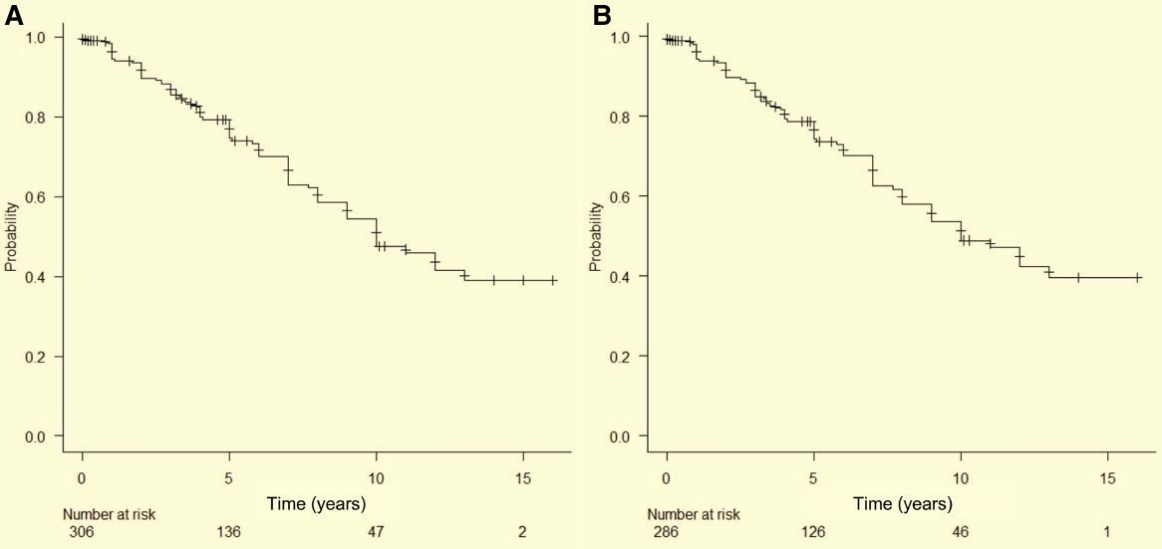
**Phenoconversion for neurodegenerative disorders in IRBD.** Rates of neurological disease-free survival using the Kaplan–Meier method, according to the time of IRBD diagnosis among the entire cohort of 306 patients (**A**) and 286 patients with IRBD and H/M ratio < 2.2 (**B**). IRBD, isolated rapid eye movement sleep behaviour disorder.

This large Japanese two-cohort study (*n* = 306) found that 91 patients (29.7%) developed an overt synucleinopathy [51 Parkinson’s disease, 35 DLB, 4 MSA (Supplementary Table 1) and 1 cerebellar ataxia] during a mean follow-up of 4.72 ± 3.94 years, with a conversion risk of 14.5% at 3 years, 25.4% at 5 years, 41.4% at 8 years and 52.5% at 10 years (Fig. 2A).

On the other hand, among patients with H/M < 2.2 in the delayed images (*n* = 286), 85 (29.7%) developed Lewy body disease (Parkinson’s disease or DLB) during a mean follow-up of 4.71 ± 3.94 years, with a conversion risk of 14.5% at 3 years, 25.6% at 5 years, 42.0% at 8 years and 51.0% at 10 years (Fig. 2B).

**Figure 3:**
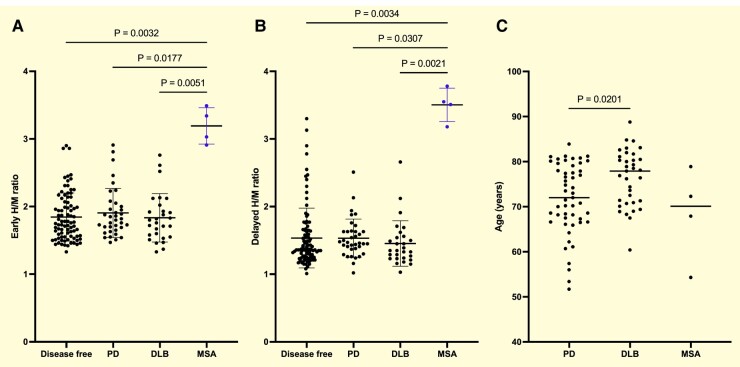
Phenoconversion age and early and delayed ^123^I-MIBG uptake at baseline in patients with IRBD. (**A**) Among IRBD patients who exhibited phenoconversion, the ^123^I-MIBG early uptake at baseline was relatively preserved in the MSA group compared to the Parkinson’s disease, DLB and disease-free groups (early image; Kruskal–Wallis test, disease free versus MSA, *P* = 0.0032; Parkinson’s disease versus MSA, *P* = 0.0177; DLB versus MSA, *P* = 0.0051, respectively). (**B**) ^123^I-MIBG delayed uptake at baseline among IRBD patients who exhibited photoconversion (disease free versus MSA, *P* = 0.0034; Parkinson’s disease versus MSA, *P* = 0.0307; DLB versus MSA, *P* = 0.0021). (**C**) Following phenoconversion from IRBD, patients with DLB at conversion were older than those with Parkinson’s disease (*P* = 0.0201).

Of the 215 patients who were followed-up until the end of the study, 51 (23.7%) developed Parkinson’s disease, 44 (86.3%) had an abnormal early ^123^I-MIBG image at baseline, and 50 (98.0%) had an abnormal delayed ^123^I-MIBG image. Of the 35 patients (16.3%) who developed DLB, 32 (91.4%) had an abnormal early ^123^I-MIBG image and 34 (97.1%) had an abnormal delayed ^123^I-MIBG image at baseline. On the other hand, of the four patients who developed MSA, all (100%) had normal uptake on both early and delayed ^123^I-MIBG images (Fig. 3A and B).

**Figure 4:**
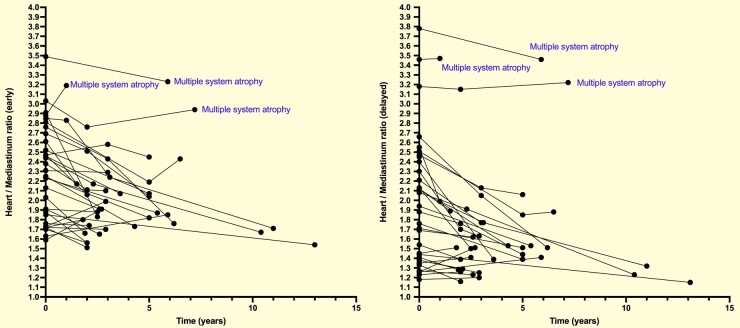
**Longitudinal assessment of ^123^I-MIBG scintigraphy in IRBD.** Repeated measurements of early and delayed ^123^I-MIBG scintigraphy in IRBD. Among the three patients who progressed to MSA, the accumulation was maintained after repeated testing from baseline. IRBD, isolated rapid eye movement sleep behaviour disorder; MSA, multiple system atrophy.

In total, 33 patients underwent ^123^I-MIBG at least twice during the follow-up, of whom 19 had normal uptake only on early images at baseline, 11 had normal uptake on both early and delayed images, and 14 had decreased uptake on both early and delayed images. During the follow-up (mean: 4.2 ± 2.8 years), the ^123^I-MIBG uptake was decreased in 16 of the 19 patients with either early or delayed normal uptake, while 3 continued to have a normal uptake. There was no significant difference in the change over time among the 14 patients with reduced uptake at baseline. Of the 30 patients with reduced uptake at baseline or during follow-up, 11 developed Parkinson’s disease, 7 progressed to DLB, and 12 remained disease free. Three cases with normal ^123^I-MIBG uptake during follow-up developed MSA (Fig. 4).

In IRBD patients, the H/M ratio for the cardiac ^123^I-MIBG uptake on both early and delayed images is weakly negatively correlated with age (Spearman rank test: *r* = −0.255, *P* < 0.001; *r* = −0. 287, *P* < 0.001, respectively), but only the H/M ratio on early images is weakly negatively correlated with disease duration (Spearman rank test: *r* = −0.39, *P* = 0.015). On the other hand, the cardiac ^123^I-MIBG uptake is weakly positively correlated with Mini-Mental Examination in terms of the H/M ratio on both early and delayed images (Spearman rank test: *r* = 0.141, *P* = 0.015; *r* = 0.195, *P* < 0.001, respectively), but is no correlated with Unified Parkinson Disease Rating Scale-III when considering the H/M ratio on both early and delayed images (Spearman rank test: *r* = −0.071, *P* = 0.220; *r* = −0.023, *P* = 0.698, respectively).

## Discussion

This study demonstrates that the cardiac ^123^I-MIBG uptake is reduced in most IRBD patients and deteriorates markedly over a 3-year period. To our knowledge, this is the first study to assess the cardiac ^123^I-MIBG progression in IRBD using imaging markers and to observe outcomes longitudinally. Our findings suggest the existence of a pattern of cardiac ^123^I-MIBG progression in IRBD. First, there is measurable degeneration of cardiac noradrenergic fibres from the time of IRBD diagnosis to disease progression among patients who develop Lewy body disease, while the uptake is preserved in patients who progress to MSA with no further decline in the cardiac ^123^I-MIBG uptake.

Among patients who progressed from IRBD to Lewy body disease (Parkinson’s disease or DLB), those who progressed to DLB were older at the time of diagnosis than those who progressed to Parkinson’s disease (Fig. 3C). Even if the incidence of both Parkinson’s disease and DLB increases with age, DLB patients are usually older than Parkinson’s disease patients at the time of diagnosis.^21^ Late-onset IRBD was associated with a higher level of neurodegenerative markers and more rapid phenoconversion, especially to probable DLB. Age at the onset of IRBD can help identify clinical features and predict the prognosis of IRBD.^22^

In a recent multicentre study from 28 centre across 12 countries, including South Korea and China, the 3-year phenoconversion rate was 18.2%^14^ compared to 14.5% in the present study. Recent studies, including our previous study from Asia, have shown lower phenoconversion rates^19^ of 7.2% at 3 years from South Korea,^23^ 4.7% at 3 years from China^24^ and 19.6% at 5 years from Turkey.^25^ These results suggest that methodological differences between studies, such as sample size, follow-up duration and definition of phenoconversion (e.g. whether or not to include mild cognitive impairment), may contribute to the different results. As a result, future studies should also consider racial and geographical differences and referral bias.^19^ This study is the first to determine the conversion rate in a homogeneous cohort with IRBD and reduced ^123^I-MIBG uptake over time. Reduced ^123^I-MIBG uptake is commonly observed in IRBD patients with moderate subjective disease, and its frequency does not significantly increase with prolonged disease duration. This finding suggests that reduced ^123^I-MIBG uptake is the earliest sign of Lewy body disease and can indicate the presence of incidental Lewy bodies.^26,27^ Among these prodromal symptoms, IRBD has a strong association with the risk of Lewy body disease, suggesting that this is a useful diagnostic tool for prodromal-stage Lewy body disease.^28^

From a pathophysiological point of view, the reduced cardiac ^123^I-MIBG uptake suggests that the abnormality is not confined to the brain, and that the sympathetic function is more extensively impaired even in the early stages of Lewy body disease.^29,30^ Indeed, Lewy bodies are widely distributed in the hypothalamus, sympathetic nervous system, parasympathetic nervous system, cardiac plexus, gastrointestinal tract enteric nervous system, pelvic plexus and adrenal medulla.^2^ From a genetic perspective, the finding that symptomatic carriers of PARK1 mutations show a similar degree of delayed H/M ratio as DLB and IRBD patients, diseases characterized by the presence of Lewy body, supports the validity of cardiac ^123^I-MIBG scintigraphy as a biomarker of Lewy body spectrum disorders. These findings are based on analysis of various idiopathic and genetic variants of Parkinson’s disease associated with varying degrees of Lewy body pathology.^31^

A previous study suggests that cardiac denervation precedes nigrostriatal damage in IRBD patients.^32-34^ Objective markers, bowel dysfunction and cardiac sympathetic denervation worsen over 3 years in the majority of IRBD patients, and the lack of correlation between markers and nigrostriatal dopaminergic dysfunction suggests that progressive gastrointestinal and cardiac dysfunction in IRBD are mainly caused by non-dopaminergic mechanisms.^35^

Prodromal MSA in IRBD progresses rapidly, often without substantial autonomic dysfunction, and olfactory and cognitive functions are maintained throughout the prodromal phase.^36^ A novel finding of our study is the normal cardiac ^123^I-MIBG uptake values in the four patients with prodromal MSA at baseline and during follow-up. Our results suggest that one pathway of prodromal MSA, which emerges from prodromal IRBD, is different from that of Lewy body disease.^37^ These findings have implications for the differentiation of early stages of MSA and for the development of diagnostic criteria for prodromal MSA. In such cases, in combination with the ^123^I-MIBG test, markers that may be used include an olfactory identification test,^38^ differential symptoms that can be used in the clinical diagnosis of MSA,^39^ stridor or sleep-disordered breathing^40^ and MRI findings^41^ (Supplementary Table 1).

The main limitations of the present study were its retrospective design and a lack of pathological data, as Alzheimer’s disease and Lewy body pathologies frequently coexist in DLB and Alzheimer’s disease.^2,42^ The abnormal findings of ^123^I-MIBG cardiac scintigraphy strongly support the presence of Lewy body disease and cardiac sympathetic denervation, which is in line with a previous clinicopathological validation study.^30^ Our repeated observations of cases suggest that cases in which ^123^I-MIBG uptake decreases over time progress to Parkinson’s disease/DLB, while cases in which uptake does not decrease over time progress to MSA. In IRBD patients with normal uptake at baseline, it is necessary to perform ^123^I-MIBG repeatedly and observe changes in the degree of ^123^I-MIBG uptake. However, normal cardiac ^123^I-MIBG uptake cannot exclude the pathogenesis of Lewy body disease, which can be more accurately diagnosed by observing changes over time or by evaluating other biomarkers.^43^ Furthermore, the timing of IRBD onset may be subject to recall bias. Based on our results, in which ^123^I-MIBG uptake was reduced in >90% of IRBD patients at the time of PSG confirmation, the timing of the initial decline in ^123^I-MIBG uptake in prodromal RBD is unclear.^44^ The future question is whether RBD-negative cases with reduced ^123^I-MIBG uptake will develop RBD or progress to neurodegenerative diseases without developing RBD. The latter scenario may affect the sensitivity of the detection of progression of IRBD with reduced ^123^I-MIBG uptake to Lewy body disease. Even at the end of the observation period in this study, cases with decreased ^123^I-MIBG uptake that are still disease free are at risk of progression to Parkinson’s disease/DLB if followed for a longer period of time. Therefore, we would expect the results to be more robust with a longer follow-up.

The main strengths of the present study are the large sample size and use of standardized post-processing of cardiac ^123^I-MIBG scintigraphy based on a widely available, registered tool in Japan. The diagnostic accuracy of IRBD was enhanced by the use of PSG. The medical records provided longitudinal neurodiagnostic follow-up data for every 1–3-month interval. A number of studies have suggested that the presence of non-motor problems, including autonomic abnormalities, such as IRBD and orthostatic hypotension, are associated with diffuse and malignant subtypes of Parkinson’s disease with high rates of progression and a high incidence of dementia. IRBD and autonomic dysfunction are strongly correlated. A major hurdle for developing and testing such curative therapies results from the fact that most dopaminergic neurons are already lost at the time of the clinical diagnosis, rendering them inaccessible to therapy. Understanding the early pathological changes that precede Lewy body pathology and cell loss in Parkinson’s disease or DLB will likely support the development of novel diagnostic and therapeutic strategies and help to differentiate Lewy body disease, non-Lewy body disease and MSA alterations. Cardiac ^123^I-MIBG is highly efficient at distinguishing at-risk individuals who are diagnosed subsequently with Lewy body disease from those who develop non-Lewy body disease or MSA. The current findings from IRBD patients suggest that the temporal changes in ^123^I-MIBG may be a potential autonomic outcome measure and a component of efficacy analysis. However, further clinical trials are warranted.

In conclusion, IRBD patients with reduced cardiac ^123^I-MIBG uptake (H/M < 2.2) are at higher risk of phenoconversion and may manifest Lewy body disease over a mean duration of 4.7 years. There was a weak trend of reduced cardiac ^123^I-MIBG uptake over age among IRBD patients. In contrast, patients with retained uptake over time and no increase in the washout rate were more likely to progress to MSA or RBD mimics. IRBD patients in whom the ^123^I-MIBG uptake does not decline during follow-up may progress to MSA. Therefore, the ^123^I-MIBG uptake, in combination with other promising biomarkers, can predict the likelihood of conversion to MSA. The present longitudinal study indicates that cardiac ^123^I-MIBG uptake is a robust biomarker for predicting the transition from IRBD to Lewy body disease. These findings can be used to stratify patients for enrolment in future neuroprotective clinical trials.

## Supplementary Material

fcae148_Supplementary_Data

## Data Availability

The data that support the findings of this study are available from the corresponding author upon reasonable request.

## References

[1] Schenck CH, Bundlie SR, Ettinger MG, Mahowald MW. Chronic behavioral disorders of human REM sleep: a new category of parasomnia. Sleep. 1986;9(2):293–308. 3505730 10.1093/sleep/9.2.293

[2] Iranzo A, Tolosa E, Gelpi E, et al Neurodegenerative disease status and post-mortem pathology in idiopathic rapid-eye-movement sleep behaviour disorder: An observational cohort study. Lancet Neurol. 2013;12(5):443–453. 23562390 10.1016/S1474-4422(13)70056-5

[3] Galbiati A, Verga L, Giora E, Zucconi M, Ferini-Strambi L. The risk of neurodegeneration in REM sleep behavior disorder: A systematic review and meta-analysis of longitudinal studies. Sleep Med Rev. 2019;43:37–46. 30503716 10.1016/j.smrv.2018.09.008

[4] Nakajima K, Nakata T. Cardiac 123I-MIBG imaging for clinical decision making: 22-year experience in Japan. J Nucl Med. 2015;56(Suppl 4):11S–19S. 26033897 10.2967/jnumed.114.142794

[5] Orimo S, Suzuki M, Inaba A, Mizusawa H. 123I-MIBG myocardial scintigraphy for differentiating Parkinson’s disease from other neurodegenerative parkinsonism: A systematic review and meta-analysis. Parkinsonism Relat Disord. 2012;18(5):494–500. 22321865 10.1016/j.parkreldis.2012.01.009

[6] Yoshita M, Arai H, Arai H, et al Diagnostic accuracy of 123I-meta-iodobenzylguanidine myocardial scintigraphy in dementia with Lewy bodies: A multicenter study. PLoS One. 2015;10(3):e0120540. 25793585 10.1371/journal.pone.0120540 PMC4368705

[7] Komatsu J, Samuraki M, Nakajima K, et al (123)I-MIBG myocardial scintigraphy for the diagnosis of DLB: A multicentre 3-year follow-up study. J Neurol Neurosurg Psychiatry. 2018;89(11):1167–1173. 29853532 10.1136/jnnp-2017-317398

[8] Choi MH, Yoon JH, Yong SW. Cardiac sympathetic denervation and dementia in de novo Parkinson’s disease: A 7-year follow-up study. J Neurol Sci. 2017;381:291–295. 28991700 10.1016/j.jns.2017.09.010

[9] Uchiyama M, Isse K, Tanaka K, et al Incidental Lewy body disease in a patient with REM sleep behavior disorder. Neurology. 1995;45(4):709–712. 7723959 10.1212/wnl.45.4.709

[10] Boeve BF, Silber MH, Saper CB, et al Pathophysiology of REM sleep behaviour disorder and relevance to neurodegenerative disease. Brain. 2007;130(Pt 11):2770–2788. 17412731 10.1093/brain/awm056

[11] Miyamoto T, Miyamoto M, Suzuki K, Nishibayashi M, Iwanami M, Hirata K. 123I-MIBG cardiac scintigraphy provides clues to the underlying neurodegenerative disorder in idiopathic REM sleep behavior disorder. Sleep. 2008;31(5):717–723. 18517041 10.1093/sleep/31.5.717 PMC2398741

[12] Miyamoto T, Miyamoto M, Inoue Y, Usui Y, Suzuki K, Hirata K. Reduced cardiac 123I-MIBG scintigraphy in idiopathic REM sleep behavior disorder. Neurology. 2006;67(12):2236–2238. 17190953 10.1212/01.wnl.0000249313.25627.2e

[13] King AE, Mintz J, Royall DR. Meta-analysis of 123I-MIBG cardiac scintigraphy for the diagnosis of Lewy body-related disorders. Mov Disord. 2011;26(7):1218–1224. 21480373 10.1002/mds.23659

[14] Joza S, Hu MT, Jung KY, et al Progression of clinical markers in prodromal Parkinson’s disease and dementia with Lewy bodies: A multicentre study. Brain. 2023;146(8):3258–3272. 36881989 10.1093/brain/awad072

[15] Gilman S, Wenning GK, Low PA, et al Second consensus statement on the diagnosis of multiple system atrophy. Neurology. 2008;71(9):670–676. 18725592 10.1212/01.wnl.0000324625.00404.15 PMC2676993

[16] Postuma RB, Berg D, Stern M, et al MDS clinical diagnostic criteria for Parkinson’s disease. Mov Disord. 2015;30(12):1591–1601. 26474316 10.1002/mds.26424

[17] McKeith IG, Boeve BF, Dickson DW, et al Diagnosis and management of dementia with Lewy bodies: Fourth consensus report of the DLB Consortium. Neurology. 2017;89(1):88–100. 28592453 10.1212/WNL.0000000000004058 PMC5496518

[18] Wenning GK, Stankovic I, Vignatelli L, et al The Movement Disorder Society criteria for the diagnosis of multiple system atrophy. Mov Disord. 2022;37(6):1131–1148. 35445419 10.1002/mds.29005 PMC9321158

[19] Miyamoto T, Miyamoto M. Phenoconversion from idiopathic rapid eye movement sleep behavior disorder to Lewy body disease. Mov Disord Clin Pract. 2018;5(5):506–511. 30515439 10.1002/mdc3.12647 PMC6207131

[20] Nakajima K, Okuda K, Yoshimura M, et al Multicenter cross-calibration of I-123 metaiodobenzylguanidine heart-to-mediastinum ratios to overcome camera-collimator variations. J Nucl Cardiol. 2014;21(5):970–978. 24942608 10.1007/s12350-014-9916-2 PMC4167440

[21] Savica R, Boeve BF, Logroscino G. Epidemiology of alpha-synucleinopathies: From Parkinson disease to dementia with Lewy bodies. Handb Clin Neurol. 2016;138:153–158. 27637957 10.1016/B978-0-12-802973-2.00009-4

[22] Zhou L, Huang B, Wang J, et al Early- and late-onset of isolated rapid eye movement sleep behavior disorder: A retrospective cohort study. Sleep Med. 2023;105:1–8. 36934616 10.1016/j.sleep.2023.03.007

[23] Hong JK, Kim JM, Kim KW, Han JW, Ahn S, Yoon IY. Clinical manifestation of patients with isolated rapid eye movement sleep behavior disorder after modest-to-long disease duration. Sleep. 2022;45(6):zsac071. 35325247 10.1093/sleep/zsac071

[24] Wing YK, Li SX, Mok V, et al Prospective outcome of rapid eye movement sleep behaviour disorder: Psychiatric disorders as a potential early marker of Parkinson’s disease. J Neurol Neurosurg Psychiatry. 2012;83(4):470–472. 22250185 10.1136/jnnp-2011-301232

[25] Evlice A, Over F, Balal M, Ates E, Aslan-Kara K. Which factors affect phenoconversion in isolated rapid eye movement sleep behavior disorder? Sleep Med. 2024;113:152–156. 38016361 10.1016/j.sleep.2023.11.023

[26] Orimo S, Takahashi A, Uchihara T, et al Degeneration of cardiac sympathetic nerve begins in the early disease process of Parkinson’s disease. Brain Pathol. 2007;17(1):24–30. 17493034 10.1111/j.1750-3639.2006.00032.x PMC8095543

[27] Orimo S, Uchihara T, Nakamura A, et al Axonal alpha-synuclein aggregates herald centripetal degeneration of cardiac sympathetic nerve in Parkinson’s disease. Brain. 2008;131(Pt 3):642–650. 18079166 10.1093/brain/awm302

[28] McKeith IG, Ferman TJ, Thomas AJ, et al Research criteria for the diagnosis of prodromal dementia with Lewy bodies. Neurology. 2020;94(17):743–755. 32241955 10.1212/WNL.0000000000009323 PMC7274845

[29] Takahashi M, Ikemura M, Oka T, et al Quantitative correlation between cardiac MIBG uptake and remaining axons in the cardiac sympathetic nerve in Lewy body disease. J Neurol Neurosurg Psychiatry. 2015;86(9):939–944. 25935891 10.1136/jnnp-2015-310686

[30] Matsubara T, Kameyama M, Tanaka N, et al Autopsy validation of the diagnostic accuracy of (123)I-metaiodobenzylguanidine myocardial scintigraphy for Lewy body disease. Neurology. 2022;98(16):e1648–e1659. 35256483 10.1212/WNL.0000000000200110 PMC9052572

[31] Gabilondo I, Llorens V, Rodriguez T, et al Myocardial MIBG scintigraphy in genetic Parkinson’s disease as a model for Lewy body disorders. Eur J Nucl Med Mol Imaging. 2019;46(2):376–384. 30324423 10.1007/s00259-018-4183-0

[32] Knudsen K, Fedorova TD, Hansen AK, et al In-vivo staging of pathology in REM sleep behaviour disorder: A multimodality imaging case-control study. Lancet Neurol. 2018;17(7):618–628. 29866443 10.1016/S1474-4422(18)30162-5

[33] Janzen A, Vadasz D, Booij J, et al Progressive olfactory impairment and cardiac sympathetic denervation in REM sleep behavior disorder. J Parkinsons Dis. 2022;12(6):1921–1935. 35754288 10.3233/JPD-223201 PMC9535565

[34] Nishikawa N, Murata M, Hatano T, et al Idiopathic rapid eye movement sleep behavior disorder in Japan: An observational study. Parkinsonism Relat Disord. 2022;103:129–135. 36113390 10.1016/j.parkreldis.2022.08.011

[35] Fedorova TD, Knudsen K, Andersen KB, et al Imaging progressive peripheral and central dysfunction in isolated REM sleep behaviour disorder after 3 years of follow-up. Parkinsonism Relat Disord. 2022;101:99–104. 35853349 10.1016/j.parkreldis.2022.07.005

[36] Postuma RB, Pelletier A, Gagnon JF, Montplaisir J. Evolution of prodromal multiple system atrophy from REM sleep behavior disorder: A descriptive study. J Parkinsons Dis. 2022;12(3):983–991. 35094998 10.3233/JPD-213039 PMC9789475

[37] Borghammer P, Horsager J, Andersen K, et al Neuropathological evidence of body-first vs. brain-first Lewy body disease. Neurobiol Dis. 2021;161:105557. 34763110 10.1016/j.nbd.2021.105557

[38] Miyamoto T, Miyamoto M. Odor identification predicts the transition of patients with isolated RBD: A retrospective study. Ann Clin Transl Neurol. 2022;9(8):1177–1185. 35767550 10.1002/acn3.51615 PMC9380141

[39] Xia C, Postuma RB. Diagnosing multiple system atrophy at the prodromal stage. Clin Auton Res. 2020;30(3):197–205. 32232688 10.1007/s10286-020-00682-5

[40] Rekik S, Martin F, Dodet P, et al Stridor combined with other sleep breathing disorders in multiple system atrophy: A tailored treatment? Sleep Med. 2018;42:53–60. 29458746 10.1016/j.sleep.2017.12.008

[41] Munoz-Lopetegi A, Berenguer J, Iranzo A, et al Magnetic resonance imaging abnormalities as a marker of multiple system atrophy in isolated rapid eye movement sleep behavior disorder. Sleep. 2021;44(1):zsaa089. 32978947 10.1093/sleep/zsaa089

[42] Boeve BF, Silber MH, Ferman TJ, et al Clinicopathologic correlations in 172 cases of rapid eye movement sleep behavior disorder with or without a coexisting neurologic disorder. Sleep Med. 2013;14(8):754–762. 23474058 10.1016/j.sleep.2012.10.015 PMC3745815

[43] Park DG, Kim JY, Kim MS, et al Neurofilament light chain and cardiac MIBG uptake as predictors for phenoconversion in isolated REM sleep behavior disorder. J Neurol. 2023;270(9):4393–4402. 37233802 10.1007/s00415-023-11785-0

[44] Hogl B, Stefani A, Videnovic A. Idiopathic REM sleep behaviour disorder and neurodegeneration - an update. Nat Rev Neurol. 2018;14(1):40–55. 29170501 10.1038/nrneurol.2017.157

