# Supplementary material for: Reduced cardiac 123I-MIBG uptake is a robust biomarker of Lewy body disease in isolated rapid eye movement sleep behaviour disorder
Source: Brain Commun. 2024 Apr 26;6(3):fcae148. doi: 10.1093/braincomms/fcae148 (PMC11081076; doi:10.1093/braincomms/fcae148)
Supplement: fcae148_Supplementary_Data [file fcae148_supplementary_data.zip › Supplementary Figure 1.pptx]

## Slide 1
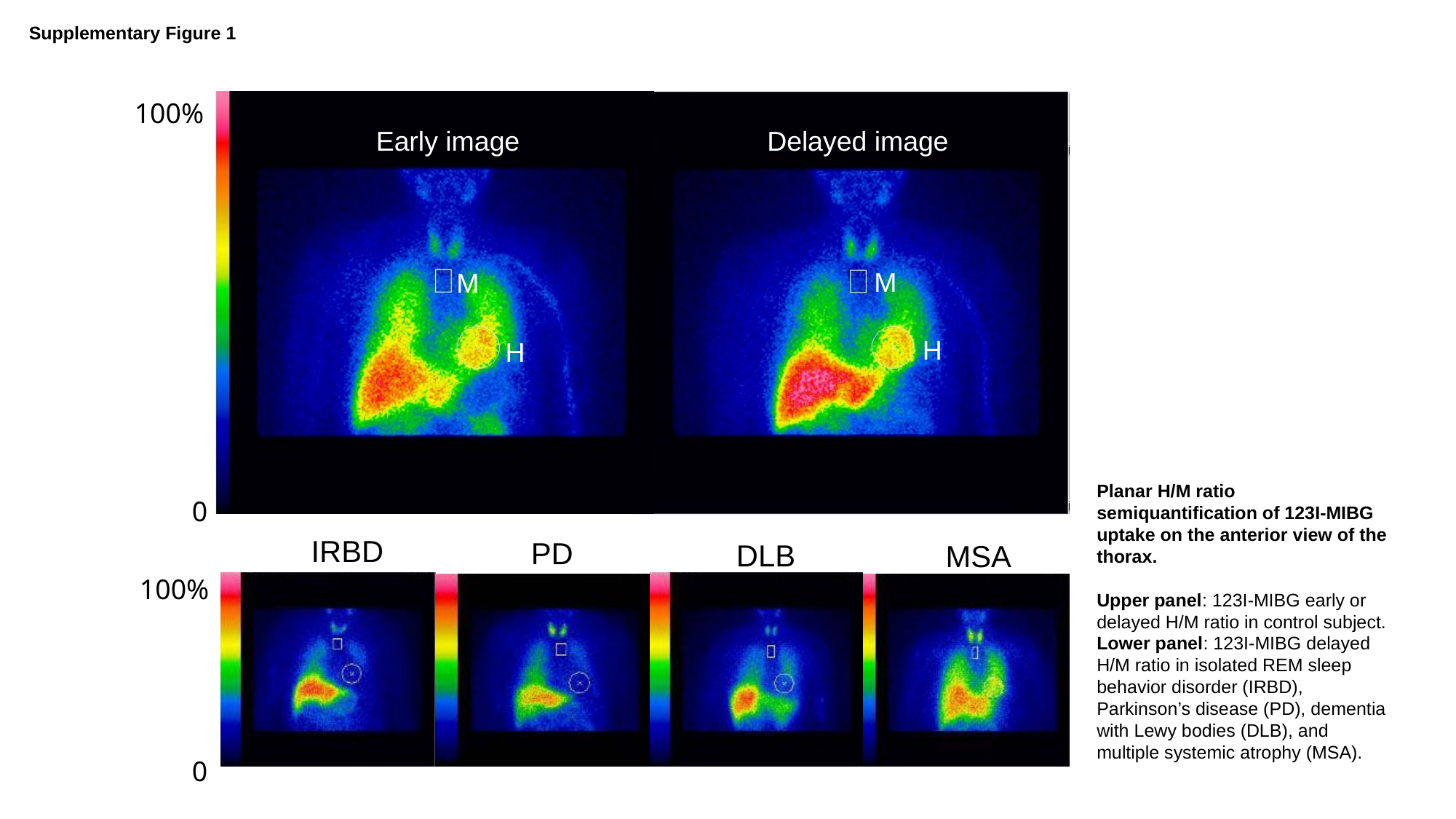

Supplementary Figure 1
100%
Early image
Delayed image
M
M
H
H
Planar H/M ratio semiquantification of 123I-MIBG uptake on the anterior view of the thorax.
Upper panel: 123I-MIBG early or delayed H/M ratio in control subject.
Lower panel: 123I-MIBG delayed H/M ratio in isolated REM sleep behavior disorder (IRBD), Parkinson’s disease (PD), dementia with Lewy bodies (DLB), and multiple systemic atrophy (MSA).
0
IRBD
PD
DLB
MSA
100%
0
