# Supplementary material for: Reduced cardiac 123I-MIBG uptake is a robust biomarker of Lewy body disease in isolated rapid eye movement sleep behaviour disorder
Source: Brain Commun. 2024 Apr 26;6(3):fcae148. doi: 10.1093/braincomms/fcae148 (PMC11081076; doi:10.1093/braincomms/fcae148)
Supplement: fcae148_Supplementary_Data [file fcae148_supplementary_data.zip › Supplementary Table 1.docx]

| **Supplementary Table 1**  **Description of baseline and diagnosed measures in patients who developed MSA** | | | | |
| --- | --- | --- | --- | --- |
| Variable | Patient 1 | Patient 2 | Patient 3 | Patient 4 |
| Age / Sex | 70 / M | 68 / M | 63 / M | 53 / F |
| Duration of RBD symptoms (y) | 9.9 | 7.3 | 8.9 | 5.3 |
| Prodromal interval MIBG baseline to MSA (y) | 8.5 | 4.6 | 4.9 | 1.5 |
| Cerebellar ataxia | No / Yes | No / Yes | No / Yes | No / Yes |
| Parkinsonism | No / No | No / Yes | No / No | No / No |
| Urinary dysfunction | No / Yes | No / Yes | No / Yes | No / Yes |
| Constipation | No / Yes | No / Yes | Yes / Yes | Yes / Yes |
| Orthostatic hypotension | Yes | N/A | Yes | No |
| Stridor | No / No | No / No | No / No | No / No |
| Laryngeal paralysis | No / No | No / No | No / No | No / No |
| SCOPA-AUT | 9 / NA | N/A / NA | N/A / NA | N/A / NA |
| Dementia | No / No | No / No | No / No | No / No |
| Clinical score |  |  |  |  |
| MMSE | 30 | 29 | 29 | 27 |
| MoCA | 30 | N/A | 24 | 25 |
| FAB | 16 | 18 | 15 | 16 |
| ESS | 8 | 5 | 14 | 8 |
| Pittsburgh sleep quality index | 3 | 4 | 4 | 5 |
| BDI II | 3 | 4 | 7 | 15 |
| UPSIT- 40 | 30 | 29 | N/A | N/A |
| UPDRS Part III | 1 | 6 | 5 | 1 |
| Apnea-Hypopnea Index ( / h) | 33.6 / NA | 26.5 / NA | 9.5 /NA | 0.4 / NA |
| RWA (%RWA, 100*RWA/REM) | Yes / NA | Yes / NA | Yes (3.66) /NA | Yes (32.21) / NA |
| MRI markers (baseline, BL / at diagnosis, Dx) | |  |  |  |
| Atrophy of | BL / Dx | BL / Dx | BL / Dx | BL / Dx |
| putamen | No / No | No / N/A | No / No | No / No |
| middle cerebellar peduncle | No / Yes | No / N/A | No / Yes | No / Yes |
| pons | No / Yes | Yes / N/A | No / Yes | No / Yes |
| cerebellum | Yes / Yes | Yes / N/A | Yes / Yes | Yes / Yes |
| "Hot cross bun" sign | No / No | No / N/A | No / No | No / Yes |
| BDI II, Beck Depression Inventory second edition; JESS, Epworth Sleepiness Scale for Japanease version; FAB, Frontal Assessment Battery; MMSE, Mini-Mental Examination; MoCA, Montreal Cognitive Assessment; MSA-C, multiple system atrophy with predominant cerebellar type; MSA-P, multiple system atrophy with predominant parkinsonism type; UPSIT, University of Pennsylvania Smell Identification Test; PSQI, Pittsburgh sleep quality index; UPDRS, Unified Parkinson Disease Rating Scale; RBD, REM sleep behavior disorder; RWA, REM sleep without atonia; SCOPA-AUT, the Scale for Outcomes of Parkinson's disease. RWA; AASM scoring Ver 2.1, surface EMG recording were chin and bilateral tibialis anterior | | | | |
